# A first report of *Thelazia callipaeda* infection in *Phortica okadai* and wildlife in national nature reserves in China

**DOI:** 10.1186/s13071-020-04509-0

**Published:** 2021-01-06

**Authors:** Yipeng Jin, Zichen Liu, Jiaqi Wei, Yifan Wen, Nianjun He, Liubin Tang, Degui Lin, Jiahao Lin

**Affiliations:** 1 College of Veterinary Medicine, People’s Republic of China Agricultural University, No. 2 Yuanmingyuan West Road, Haidian District, Beijing, 100193 People’s Republic of China; 2 Foping National Nature Reserve, Shaanxi, 723400 People’s Republic of China

**Keywords:** *Thelazia callipaeda*, *Phortica okadai*, Wildlife, Vector-borne zoonosis, Nature reserves, China

## Abstract

**Background:**

*Thelazia callipaeda* is a zoonotic parasitic nematode of the family Thelaziidae, with *Phortica okadai* as its intermediate host and only confirmed vector in China. China has the largest number of human cases of thelaziosis in the world. It is generally believed that infected domestic animals (dogs and cats) are the most important reservoir hosts of *T. callipaeda*, and thus pose a direct threat to humans. At present, there is little research or attention focused on the role of wildlife in the transmission cycle of thelaziosis in nature reserves.

**Methods:**

We selected locations in four national nature reserves across China to monitor *P. okadai* and wildlife. We used a fly-trap method to monitor *P. okadai* density. Morphological analysis of the parasites collected from the conjunctival sac of the infected wildlife was undertaken as the first step in species identification, and polymerase chain reaction (PCR) was used for species confirmation.

**Results:**

In 2019, the density of *P. okadai* in Foping National Nature Reserve in China increased sharply, and infected *P. okadai* were newly found in the reserve. Giant panda, wild boar, leopard cat, and black bear were found to be newly infected with *T. callipaeda* (one individual of each species). A total of four worms were collected, one from each species of wildlife. The four worms were identified as *T. callipaeda* by their morphological characteristics; species identification was confirmed by PCR amplification.

**Conclusions:**

To the best of our knowledge, this is the first report of *T. callipaeda* infection in *P. okadai* as well as in a variety of wildlife, including giant panda, in nature reserves in China. These results indicate that there is a transmission cycle of *T. callipaeda* among wildlife in these nature reserves. The increasing number of case reports of thelaziosis in wildlife suggest a likely risk of *T. callipaeda* infection for the inhabitants of villages situated around nature reserves. 
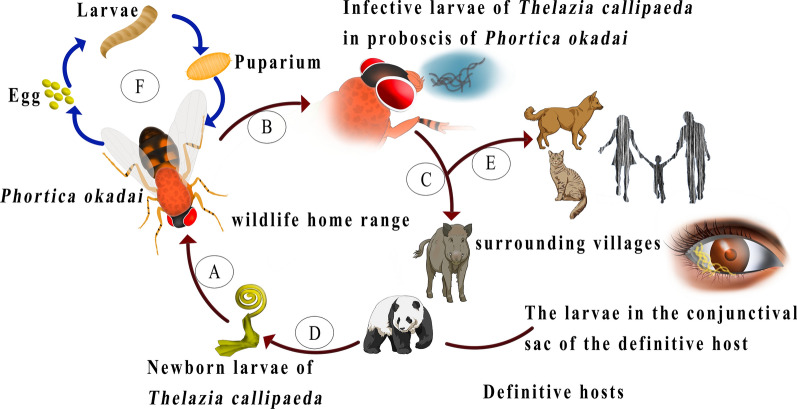

## Background

*Thelazia callipaeda* is a zoonotic parasitic nematode of the family Thelaziidae, with *Phortica okadai* (Diptera, Drosophilidae, Steganinae) as its intermediate host and only confirmed vector in China [1–5]. The definitive host of *T. callipaeda* varies widely, and includes wildlife, domestic animals and humans [6]. Dogs are its most important reservoir host. *T. callipaeda* parasitizes the conjunctival sac and lacrimal duct of mammals. Its reproductive mode is ovoviparous [7]. Infective larvae of *T. callipaeda* escape from *P. okadai*'s proboscis when the insect licks the eye of a mammal and invade the conjunctival sac [4]. The damage caused by parasitic *T. callipaeda* to the eyes of animals can differ in severity. The host’s eye can be damaged by the friction of the sharp folds of the surface of the worm and adsorption of the mouth sac by the eye tissue. In addition, adult worm secretions and excreta stimulate the tissues of the eye. During clinical examination, some infected dogs showed foreign body sensation, increased ocular secretion, eyelid edema, conjunctival congestion, inflammation or formation of small ulcers, turbidity of the aqueous humour, and increased intraocular pressure [7, 8].

In recent years, many countries have reported cases of humans infected with *T. callipaeda*, and there have also been reports of dogs and wild host animals infected with *T. callipaeda* in Italy, Germany, Serbia and other European countries [9–11]. From 1917 to 2019, a total of 643 human cases of thelaziosis were recorded in China [12, 13]. China has the largest number of human cases of thelaziosis in the world, and saw a significant increase in cases between 1970 and 1999. Although the number of human cases of thelaziosis has decreased in the past 20 years, it is starting to increase again [12, 13]. An increase in the number of *P. okadai* coupled with the proliferation of domestic animals (dogs and cats) has led to increased vigilance towards *T. callipaeda* infection.

The *T. callipaeda* infection rate of villagers is higher than that of urban dwellers [13] because the village environment is more suitable for the survival of *P. okadai*. We performed ocular examinations of domestic animals (dogs) from 2016 to 2019 in the villages around national nature reserves and found that, in 2019, the prevalence rate of thelaziosis was as high as 84.62% (88/104), which was higher than that in 2016 (38.05%, 43/113), 2017 (53.92%, 55/102) and 2018 (56.25%, 63/112).

Most nature reserves in China are surrounded by villages, and the number of wild animals in the reserves far exceeds the number of domestic animals in the villages. Thus, if the wildlife spread *T. callipaeda* on a large scale, they could pose a threat to the inhabitants of nearby villages and their domestic animals.

We selected four national nature reserves across China to monitor the prevalence of *T. callipaeda* in *P. okadai* and wildlife between 2016 and 2019. The four monitoring locations were located in the home range of the giant panda, one of the most complex topographical regions in the world, with clear vertical zoning of climate, and home to more than 8000 confirmed species of wildlife and plants. In the villages around these reserves, some villagers own domestic animals. These locations were selected for this study as the home ranges of the wildlife of the reserves and the domestic animals (dogs and cats) of the surrounding villages overlap within them [14]. The reserves were also selected because they are close to densely populated cities.

## Methods

### Monitoring locations for *P. okadai* and wildlife

**Fig. 1:**
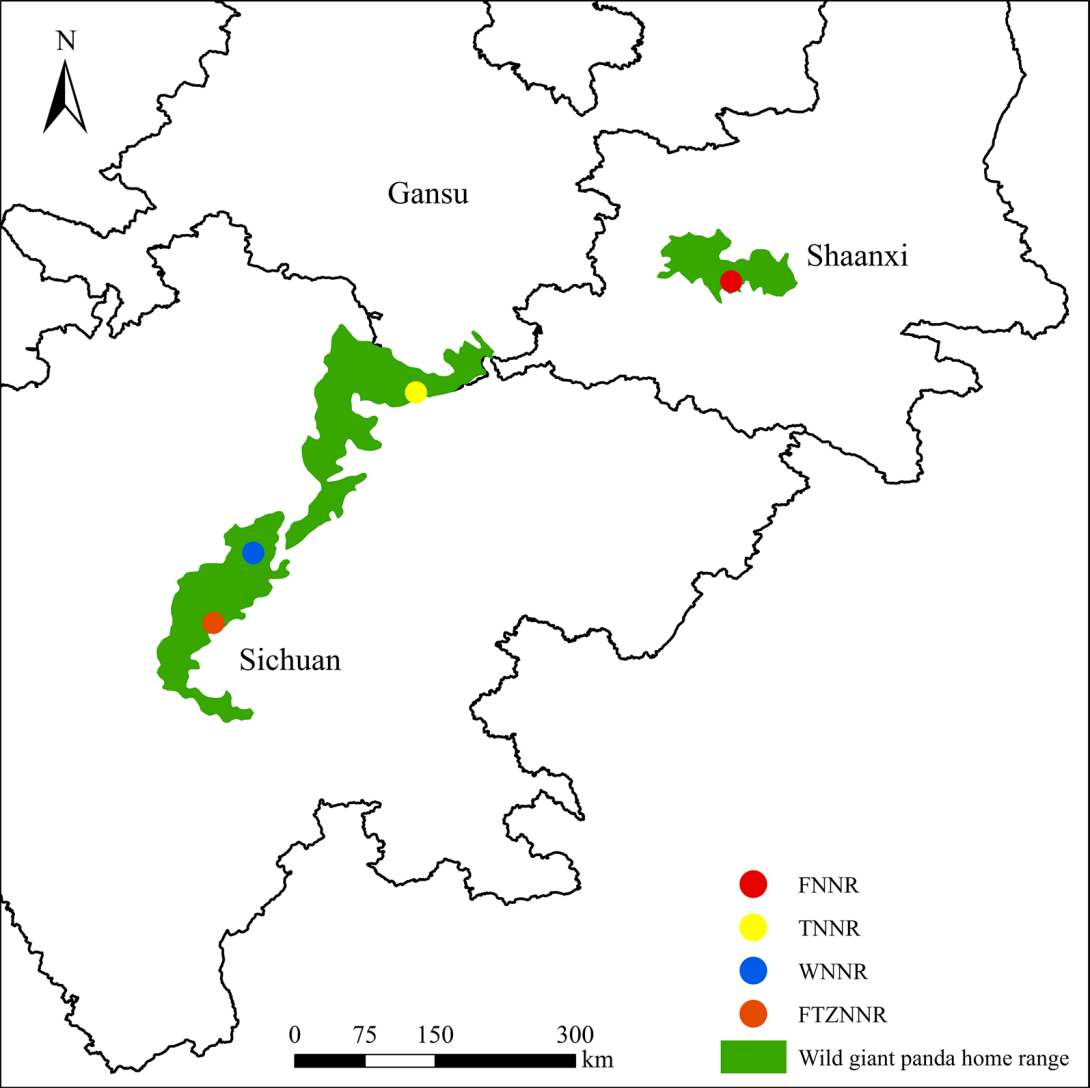
Map showing the monitoring locations for *Phortica okadai* and wildlife in the four national nature reserves between 2016 and 2019. *FNNR* Foping National Nature Reserve, *TNNR* Tangjiahe National Nature Reserve, *WNNR* Wolong National Nature Reserve, *FTZNNR* Fengtongzhai National Nature Reserve

We selected locations in the following four national nature reserves, which are situated across China, for the monitoring of *P. okadai* and wildlife between 2016 and 2019: Foping National Nature Reserve, Shaanxi (FNNR; 33°38′43"N, 107°47′38"E); Tangjiahe National Nature Reserve, Sichuan (32°34′44"N, 104°45′43"E); Wolong National Nature Reserve, Sichuan (WNNR; 31°02′20"N, 103°11′52"E); and Fengtongzhai National Nature Reserve, Sichuan (30°22′05"N, 102°48′52"E) (Fig. 1).

### Monitoring *P. okadai* density and morphological identification

In accordance with the surveillance methods for vector density—fly promulgated by the National Health Commission of the People’s Republic of China [15], we used a fly-trap method to monitor *P. okadai* density. A total of 200 traps were placed at each monitoring location, of which 100 were distributed in the home ranges of wildlife and the other 100 in the surrounding villages. The traps were baited with prepared fruit mash. Monitoring was done from April to October. The fly traps were placed at the monitoring locations at 9 a.m. in the middle of each month, and were retrieved at 9 a.m. on the following day. Because many kinds of fly were caught in the traps, *P. okadai* were first identified according to morphological characteristics. They were then removed from the traps for further morphological analysis under a microscope. The density of *P. okadai* (number per trap per 24 h) was calculated and the infection rate of *T. callipaeda* determined by dissection [15].

### Sample collection

In 2019, four cases of ocular worm infection were identified in FNNR, one in giant panda (Additional file 1: Figure S1), one in wild boar, one in leopard cat and one in black bear. Infection was determined by ophthalmic examination of the animals under anesthesia in FNNR; anesthetic was given by injection. A total of four worms were collected, one from each animal.

### Parasite collection and treatment

The worms were removed from the conjunctival sac and placed in a sampling tube containing 70% ethanol. Morphological analysis of the parasites was the first step in species identification; PCR was used for species confirmation.

### Morphological analysis

The parasites collected from the eyes of each animal were examined under a light microscope combined with a camera and identified on the basis of their morphology by using taxonomic keys.

### Sequence and phylogenetic analysis

**Fig. 2:**
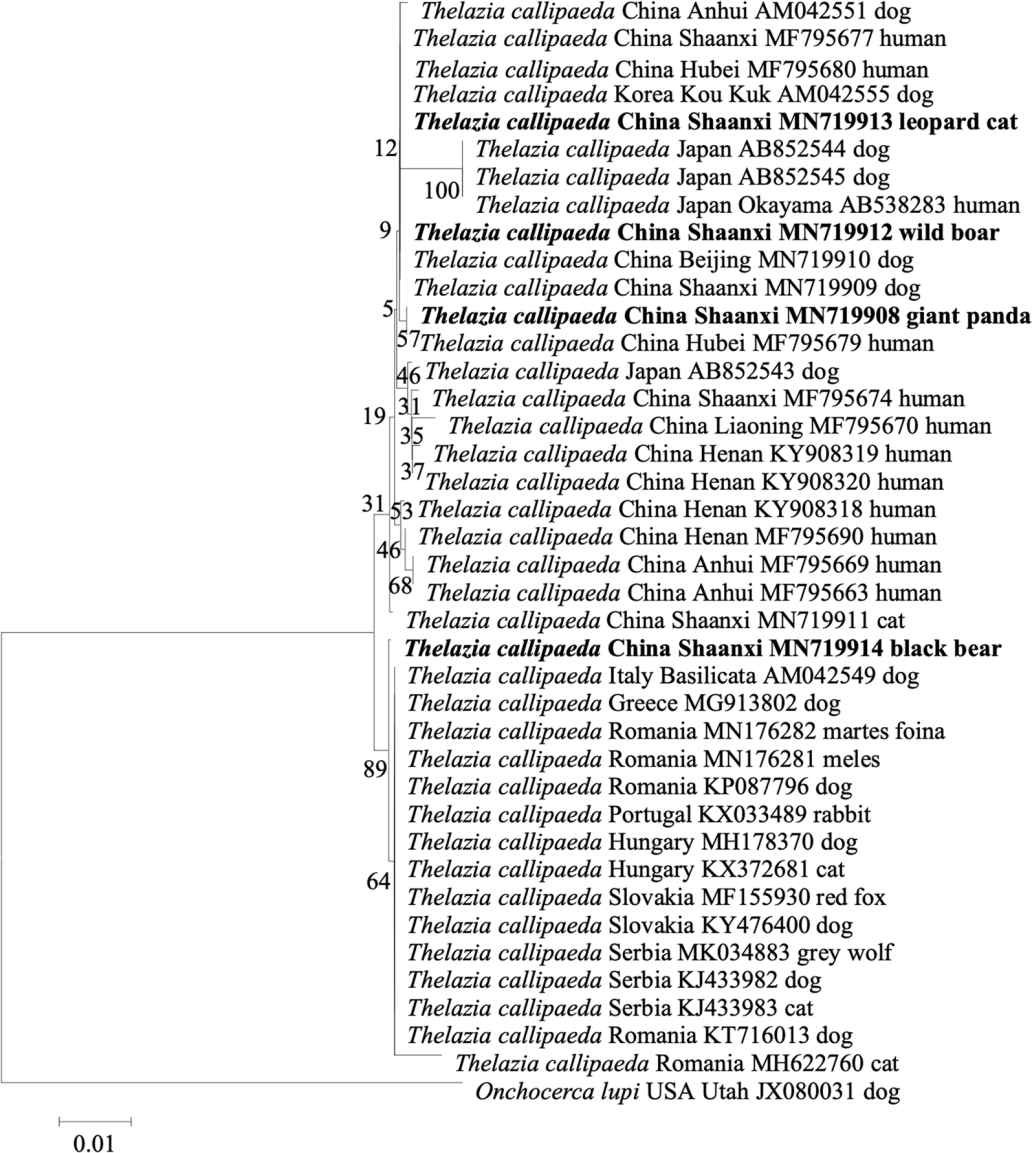
Phylogenetic tree of a partial sequence of the mitochondrial cytochrome c oxidase subunit 1 gene of *Thelazia callipaeda*. Bootstrap values are shown* next to branches*. The isolates sequenced in this study are in* bold* (GenBank accession nos. MN719908, MN719912, MN719913 and MN719914). The remaining sequences of *T. callipaeda* were downloaded from the National Center for Biotechnology Information website.* Scale bar* indicates nucleotide substitutions per site

We extracted genomic DNA of each worm from the conjunctival sac of the giant panda, wild boar, leopard cat, and black bear with the HiPure Tissue & Blood DNA Kit (MAGEN, China). A partial sequence of the mitochondrial cytochrome c oxidase subunit 1 gene (*cox*1; 689 base pairs) was amplified by PCR. Amplicons were purified by a HiPure Gel Pure Micro Kit (MAGEN, China) and sequenced in an ABI3730XL with the BigDyeTr v3.1 Cycle Seq Kit (Applied Biosystems, USA). Amplicon sequences were determined in both directions (GenBank accession nos. MN719908, MN719912, MN719913 and MN719914) and genetic analyses performed using available sequences of related nematodes from GenBank and the Global Initiative on Sharing all Influenza Data (GISAID) database (https://www.gisaid.org). The phylogenetic tree (Fig. 2) was constructed by MEGA version 6 (https://www.megasoftware.net) using the neighbor-joining method with 1000 bootstrap replicates [9, 10, 16, 17].

## Results

Examination of the worms under a microscope (Additional file 1: Figure S2) showed characteristic morphological features of *P. okadai* [2, 5, 18]; the genitalia were not examined.

**Fig. 3:**
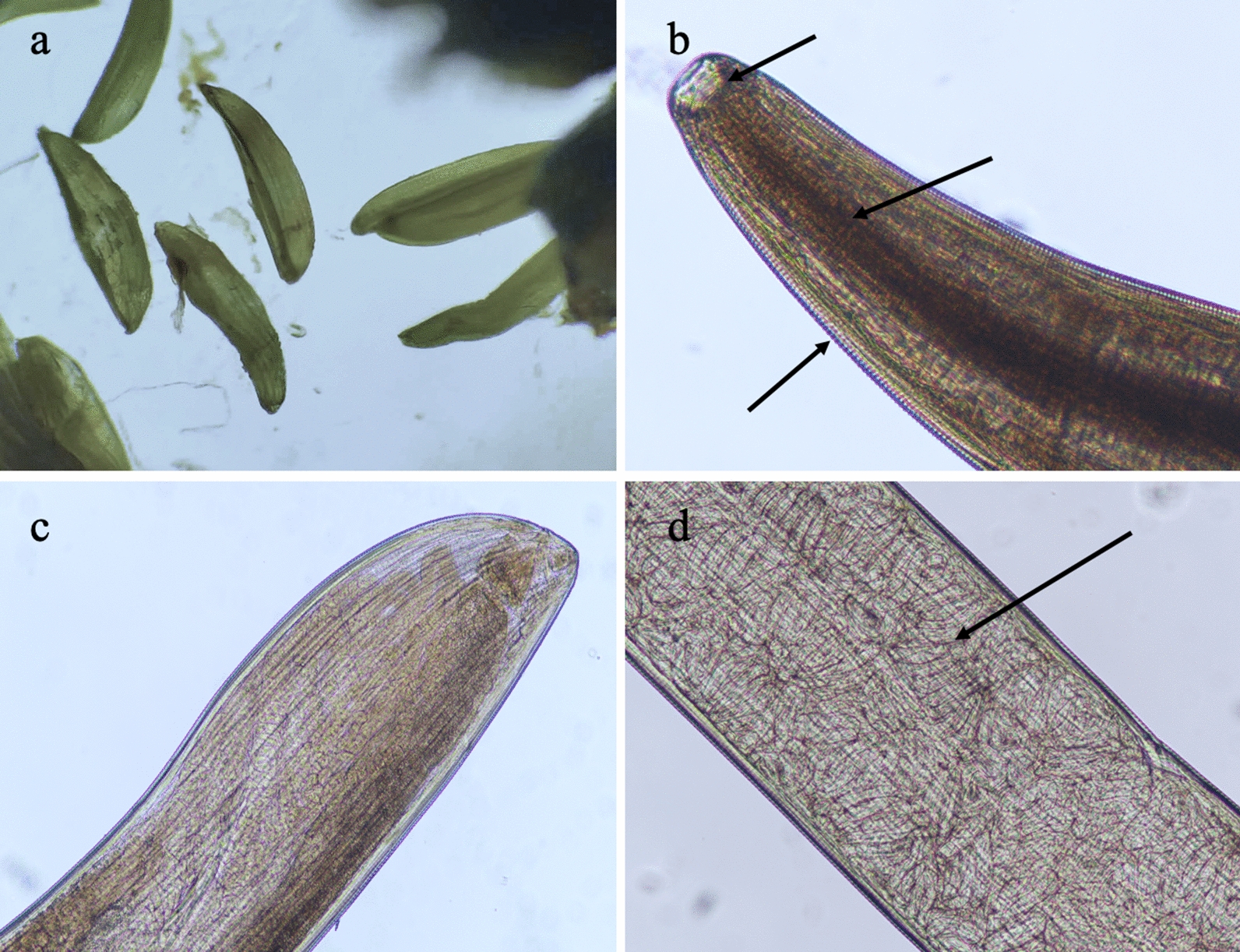
**a** Early stage pre-infective larva of *T. callipaeda* found in the haemocoel of *P. okadai*. **b** Image of *T. callipaeda* showing the buccal capsule, pharynx, and esophagus of the anterior segment of the worm body and its serrated, wrinkled surface (*arrow*). **c** Caudal end of the worm. **d** Larvae in a female worm’s uterus (*arrow*)

Before 2019 (in 2016, 2017 and 2018), there were fewer *P. okadai* in the wildlife home ranges than in the surrounding villages; no infected *P. okadai* were found in the wildlife home ranges during this period (Tables 1, 2, 3). A high number of infected *P. okadai* were found in the surrounding villages during this time (Tables 1, 2, 3). In 2019, the density of *P. okadai* increased sharply in FNNR, and infected *P. okadai* were newly found in the home ranges of the reserve’s wildlife (Table 4). The distribution density of *P. okadai* was highest in July and August, and the highest numbers of *P. okadai* infected with larvae of *T. callipaeda* were also found in these months (Fig. 3a).

**Table 1:** The density and infection situation of *Phortica okadai* in wildlife home range and surrounding villages, China, 2016

Monitoring points	Region	Months	No. Fly Traps		No. *P. okadai*		Fly density (fly/cage /24h)		No. infected *P. okadai*	
			Surrounding villages	Wildlife home range	Surrounding villages	Wildlife home range	Surrounding villages	Wildlife home range	Surrounding villages	Wildlife home range
FNNR	Shaanxi	April	100	100	141	85	1.41	0.85	69	0
		May			203	96	2.03	0.96	118	0
		June			202	101	2.02	1.01	111	0
		July			206	123	2.06	1.23	112	0
		August			221	124	2.21	1.24	126	0
		September			218	119	2.18	1.19	102	0
		October			137	113	1.37	1.13	104	0
TNNR	Sichuan	April	100	100	93	67	0.93	0.67	43	0
		May			102	81	1.02	0.81	67	0
		June			109	66	1.09	0.66	75	0
		July			114	76	1.14	0.76	87	0
		August			123	79	1.23	0.79	101	0
		September			103	65	1.03	0.65	79	0
		October			96	54	0.96	0.54	33	0
WNNR	Sichuan	April	100	100	0	0	0	0	0	0
		May			0	0	0	0	0	0
		June			0	0	0	0	0	0
		July			0	0	0	0	0	0
		August			0	0	0	0	0	0
		September			0	0	0	0	0	0
		October			0	0	0	0	0	0
FTZNNR	Sichuan	April	100	100	86	45	0.86	0.45	32	0
		May			85	33	0.85	0.33	66	0
		June			98	75	0.98	0.75	34	0
		July			103	88	1.03	0.88	79	0
		August			106	86	1.06	0.86	98	0
		September			101	76	1.01	0.76	77	0
		October			91	23	0.91	0.23	34	0

*FNNR* Foping National Nature Reserve, *TNNR*, Tangjiahe National Nature Reserve, *WNNR* Wolong National Nature Reserve, *FTZNNR* Fengtongzhai National Nature Reserve, No. Fly Traps, mean the total number of traps placed at monitoring point; No. *P. okadai* , mean the total number of *Phortica okadai* collected in the 24 h of sampling; Fly density (fly/cage /24 h), mean the number of *Phortica okadai *per cage for 24 h; No. infected *P. okadai*, mean the number of contain early stage of pre-infection larva of *Thelazia callipaeda* in fly haemocoel

**Table 2:** The density and infection situation of *Phortica okadai* in wildlife home range and surrounding villages, China, 2017

Monitoring points	Region	Months	No. Fly Traps		No. *P. okadai*		Fly density (fly/cage /24h)		No. infected *P. okadai*	
			Surrounding villages	Wildlife home range	Surrounding villages	Wildlife home range	Surrounding villages	Wildlife home range	Surrounding villages	Wildlife home range
FNNR	Shaanxi	April	100	100	181	81	1.81	0.81	90	0
		May			221	88	2.21	0.88	121	0
		June			238	103	2.38	1.03	143	0
		July			241	113	2.41	1.13	213	0
		August			272	129	2.72	1.29	222	0
		September			203	109	2.03	1.09	107	0
		October			180	99	1.80	0.99	111	0
TNNR	Sichuan	April	100	100	103	78	1.03	0.78	52	0
		May			116	86	1.16	0.86	56	0
		June			105	76	1.05	0.76	79	0
		July			124	72	1.24	0.72	94	0
		August			113	89	1.13	0.89	79	0
		September			97	71	0.97	0.71	59	0
		October			92	62	0.92	0.62	31	0
WNNR	Sichuan	April	100	100	0	0	0	0	0	0
		May			0	0	0	0	0	0
		June			0	0	0	0	0	0
		July			0	0	0	0	0	0
		August			0	0	0	0	0	0
		September			0	0	0	0	0	0
		October			0	0	0	0	0	0
FTZNNR	Sichuan	April	100	100	90	56	0.90	0.56	42	0
		May			81	32	0.81	0.32	55	0
		June			93	79	0.93	0.79	32	0
		July			112	82	1.12	0.82	68	0
		August			121	96	1.21	0.96	93	0
		September			110	74	1.10	0.74	66	0
		October			81	33	0.81	0.33	39	0

*FNNR* Foping National Nature Reserve, *TNNR* Tangjiahe National Nature Reserve, *WNNR* Wolong National Nature Reserve, *FTZNNR* Fengtongzhai National Nature Reserve; No. Fly Traps, mean the total number of traps placed at monitoring point; No. *P. okadai* , mean the total number of *Phortica okadai* collected in the 24h of sampling; Fly density (fly/cage /24 h), mean the number of *Phortica okadai* per cage for 24 h; No. infected *P. okadai*, mean the number of contain early stage of pre-infection larva of *Thelazia callipaeda* in fly haemocoel

**Table 3:** The density and infection situation of *Phortica okadai* in wildlife home range and surrounding villages, China, 2018

Monitoring points	Region	Months	No. Fly Traps		No. *P. okadai*		Fly density (fly/cage /24 h)		No. infected *P. okadai*	
			Surrounding villages	Wildlife home range	Surrounding villages	Wildlife home range	Surrounding villages	Wildlife home range	Surrounding villages	Wildlife home range
FNNR	Shaanxi	April	100	100	180	92	1.80	0.92	91	0
		May			219	79	2.19	0.79	106	0
		June			205	131	2.05	1.31	129	0
		July			212	126	2.12	1.26	209	0
		August			284	123	2.84	1.23	223	0
		September			232	116	2.32	1.16	116	0
		October			149	83	1.49	0.83	109	0
TNNR	Sichuan	April	100	100	108	61	1.08	0.61	64	0
		May			121	71	1.21	0.71	63	0
		June			112	82	1.12	0.82	73	0
		July			132	89	1.32	0.89	85	0
		August			129	92	1.29	0.92	84	0
		September			82	83	0.82	0.83	67	0
		October			99	78	0.99	0.78	43	0
WNNR	Sichuan	April	100	100	0	0	0	0	0	0
		May			0	0	0	0	0	0
		June			0	0	0	0	0	0
		July			0	0	0	0	0	0
		August			0	0	0	0	0	0
		September			0	0	0	0	0	0
		October			0	0	0	0	0	0
FTZNNR	Sichuan	April	100	100	53	31	0.53	0.31	26	0
		May			83	53	0.83	0.53	42	0
		June			62	56	0.62	0.56	33	0
		July			124	72	1.24	0.72	71	0
		August			119	89	1.19	0.89	88	0
		September			102	88	1.02	0.88	71	0
		October			92	66	0.92	0.66	40	0

*FNNR* Foping National Nature Reserve, *TNNR* Tangjiahe National Nature Reserve, *WNNR* Wolong National Nature Reserve, *FTZNNR* Fengtongzhai National Nature Reserve; No. Fly Traps, mean the total number of traps placed at monitoring point; No. *P. okadai* , mean the total number of *Phortica okadai* collected in the 24 h of sampling; Fly density (fly/cage /24 h), mean the number of *Phortica okadai *per cage for 24 h; No. infected *P. okadai*, mean the number of contain early stage of pre-infection larva of *Thelazia callipaeda* in fly haemocoel

**Table 4:** The density and infection situation of *Phortica okadai* in wildlife home range and surrounding villages, China, 2019

Monitoring points	Region	Months	No. Fly Traps		No. *P. okadai*		Fly density (fly/cage /24 h)		No. infected *P. okadai*	
			Surrounding villages	Wildlife home range	Surrounding villages	Wildlife home range	Surrounding villages	Wildlife home range	Surrounding villages	Wildlife Home Range
FNNR	Shaanxi	April	100	100	198	197	1.98	1.97	101	115
		May			201	276	2.01	2.76	166	185
		June			225	224	2.25	2.24	186	166
		July			278	256	2.78	2.56	212	206
		August			266	299	2.66	2.99	202	218
		September			223	245	2.23	2.45	167	191
		October			187	198	1.87	1.98	101	142
TNNR	Sichuan	April	100	100	123	44	1.23	0.44	67	0
		May			102	82	1.02	0.82	55	0
		June			101	99	1.01	0.99	72	0
		July			115	83	1.15	0.83	71	0
		August			113	91	1.13	0.91	78	0
		September			79	74	0.79	0.74	41	0
		October			87	76	0.87	0.76	59	0
WNNR	Sichuan	April	100	100	0	0	0	0	0	0
		May			0	0	0	0	0	0
		June			0	0	0	0	0	0
		July			0	0	0	0	0	0
		August			0	0	0	0	0	0
		September			0	0	0	0	0	0
		October			0	0	0	0	0	0
FTZNNR	Sichuan	April	100	100	61	49	0.61	0.49	39	0
		May			78	67	0.78	0.67	48	0
		June			59	48	0.59	0.48	46	0
		July			109	84	1.09	0.84	62	0
		August			106	83	1.06	0.83	64	0
		September			99	52	0.99	0.52	32	0
		October			88	51	0.88	0.51	27	0

*FNNR* Foping National Nature Reserve, *TNNR* Tangjiahe National Nature Reserve, *WNNR* Wolong National Nature Reserve, *FTZNNR* Fengtongzhai National Nature Reserve, No. Fly Traps mean the total number of traps placed at monitoring point; No. *P. okadai* , mean the total number of *Phortica okadai* collected in the 24 h of sampling; Fly density (fly/cage /24 h), mean the number of *Phortica okadai *per cage for 24 h; No. infected *P. okadai*, mean the number of contain early stage of pre-infection larva of *Thelazia callipaeda* in fly haemocoel

Morphological observation of the collected parasites showed that they had a cylindrical shape with thin ends, and were milky white in color and slightly transparent. All of the worms were female. The mean body length and width of the females were 13.7 mm and 0.36 mm, respectively. The buccal capsule, pharynx, and esophagus of the anterior segment of the worm body, its serrated, wrinkled surface and the caudal end of the worm were visible under the optical microscope (Fig. 3b, c). Larvae and oval shaped eggs were found in the mid-section of the uterus (Fig. 3d). The worms were identified as *T. callipaeda* from their morphological characteristics.

**Fig. 4:**
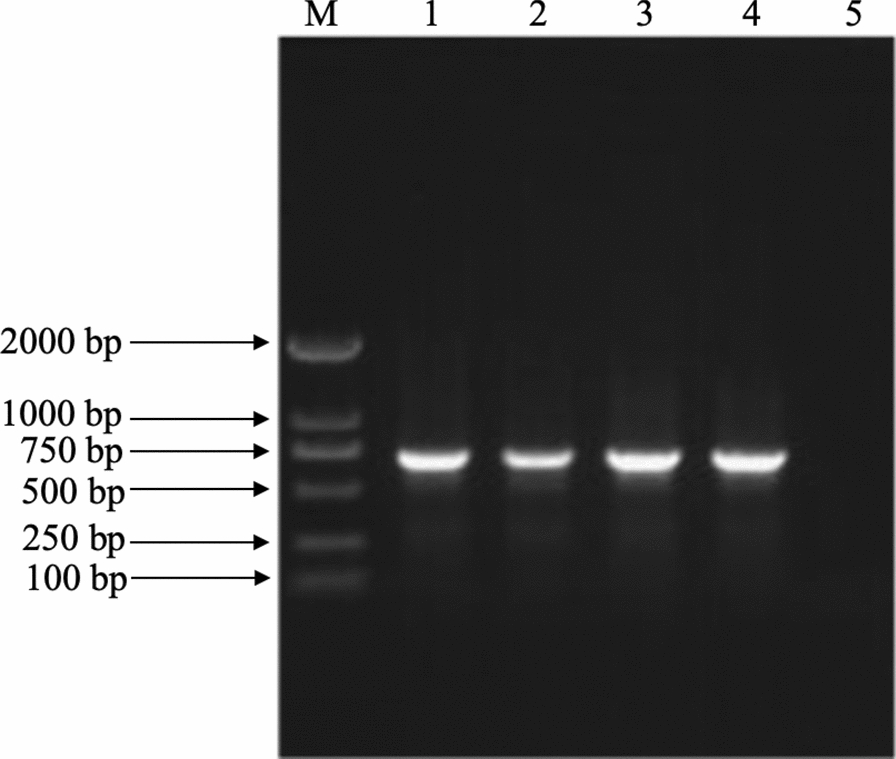
Gel electrophoresis map of polymerase chain reaction-amplified sequences of *cox*1 of *T. callipaeda*. *M* Marker, *1 cox*1-positive products of* T. callipaeda* from giant panda, *2 cox*1-positive products of* T. callipaeda* from wild boar, *3 cox*1-positive products of* T. callipaeda* from leopard cat, *4 cox*1-positive products of* T. callipaeda* from black bear, *5* negative control.* bp* Base pairs

The 2% agarose gel electrophoresis of the PCR amplified products of each sample showed a DNA band of about 689 base pairs in length, which was consistent with the expected size of the *T. callipaeda cox*1 sequence (Fig. 4). There were no non-specific bands. The alignment and phylogenetic analyses of the neighbor-joining method showed that the *T. callipaeda cox*1 sequences of giant panda, wild boar and leopard cat (GenBank accession nos. MN719908, MN719912 and MN719913) clustered with those of *T. callipaeda* in dogs, cats, and humans from China, Japan and Korea. The sequences from the black bear (GenBank accession nos. MN719914) were closely related to those from European animals, as they were clustered together. All the *cox*1 sequences of *T. callipaeda* clustered into one large branch, while the *cox*1 sequences of *Onchocerca lupi* from the USA formed an outgroup (Fig. 2).

We can confirm from morphological observations and *cox*1 detection that the ocular worms, of which there were four in total, collected from a giant panda (Additional file 1: Figure S1), wild boar, leopard cat and black bear (one individual of each species) were *T. callipaeda*.

## Discussion

It is generally believed that domestic animals are the most important reservoir hosts of *T. callipaeda*, and thus pose a direct threat to humans [6, 8, 11, 19–21]. However, in the present study, we identified cases of thelaziosis in giant panda, wild boar, leopard cat, and black bear in 2019. In the same year, a large number of the intermediate host, *P. okadai*, were also found to be infected in the home ranges of wildlife in FNNR. Thus, we believe that thelaziosis has already spread in this nature reserve. The outbreak of *T. callipaeda* infections in *P. okadai* within the home ranges of wildlife in FNNR shows that the transmission cycle of this parasite has been established there.

Summer is the most active season for the intermediate host of *T. callipaeda* [22], and is also the season in which numerous wildlife species migrate in nature reserves [14, 23–25]. Newborn larvae of *T. callipaeda* are produced directly in the conjunctival sac of infected wildlife. When the intermediate host, *P. okadai*, licks the secretions of an infected animals’s eye, it sucks the newborn larvae into its body. After ecdysis of the larvae, which occurs twice, they develop into infective larvae, at which point they enter the head and proboscis of the *P. okadai*. When the *P. okadai* then licks the eye of another wildlife host, the infective larvae of *T. callipaeda* escape from the proboscis and invade the new host’s conjunctival sac. It usually takes 15–20 days for a larva to develop into an adult [7]. Female *T. callipaeda* can continue to produce larvae until they are about 35 days old; these larvae are then licked from the host’s eye by the intermediate host, *P. okadai*, and thus the cycle continues. Thus, *P. okadai* infected with *T. callipaeda* pose a threat to both domestic animals and inhabitants of the villages surrounding the nature reserves.

Once thelaziosis is widespread among the wildlife of the reserves, villagers in the surrounding villages will inevitably be susceptible to infection due to their frequent activities near the home ranges of the wildlife. If thelaziosis is not controlled, the infected wildlife will gradually form a bank of reservoir hosts among the wildlife of the reserves. As winter approaches, wildlife migrate to the villages, which leads to interactions between wildlife and domestic animals [14, 23–25]. These interactions create an opportunity for the transmission of *T. callipaeda* between wildlife and domestic animals by *P. okadai*.

FNNR had the highest density of *P. okadai* compared to the other studied biotopes, due to its geography and climate [22]. FNNR is located in Foping county, northeast Hanzhong city, Shaanxi Province, within the northern sub-tropical climate zone. The Qinling mountains to the north of FNNR and Daba mountains to the south, which have an altitude range of 980-2904 m, form two natural barriers. As humid air does not spread easily northwards, the climate of FNNR is mild and humid. This climate is suitable for the vector of *T. callipaeda*, *P. okadai*. *P. okadai* was not found in the WNNR monitoring points. The reason for this is that most of the mountain peaks there are over 4000 m in altitude, and the average annual temperature is 8.5±0.5 ℃, which seems to be too low for the survival of *P. okadai*.

In this study, only four *T. callipaeda* were collected in total, i.e. an individual worm from four species of wildlife. The animals had to be anesthetized for parasite collection, and sampling was extremely problematic due to the large home ranges and irregular movements of the animals. Therefore, to better elucidate the actual number of animals and species of wildlife infected, we need to continue to sample and screen. 

We selected a partial sequence of the mitochondrial* cyt*1 gene as a genetic marker for the phylogenetic analysis of the parasites collected from the eyes of the infected animals. Because of its small molecular weight and strongly conserved structure, many researchers consider that *cyt*1 can be used as a specific genetic marker for some nematodes. As early as 2005, Dominico Otranto and coworkers successfully analyzed the genetic variability of *T. callipaeda* from Europe and Asia by sequencing and mutation scanning the mitochondrial *cyt*1 [17]. Therefore, we believe that using *cox*1 to reveal the genetic relationships and molecular evolution of *T. callipaeda* is extremely promising.

Although no cases of thelaziosis were found in wildlife in the three other reserves included in this study, we believe that continuous monitoring of the intermediate host needs to be carried out in these and in FNNR. In addition, domestic animals in the surrounding villages should be given deworming medicine regularly. These efforts would be helpful in monitoring the epidemiology of thelaziosis and weakening its threat to the public.

## Conclusions

To the best of our knowledge, this is the first report of *T. callipaeda* infection in *P. okadai*, as well in a variety of wildlife, including giant panda, in nature reserves in China. The results of this study illustrate the important role of wildlife in vector-borne zoonoses. Further studies are needed to undertake risk assessments of *T. callipaeda* infection in the villagers who live around these nature reserves.

## Supplementary Information

**Additional file 1: Figure S1.***Thelazia callipaeda* in the eye of a giant panda. **Figure S2**. Light micrographs of *Phortica okadai*. **a** White band around the compound eye (*arrow*); **b** multiple dark brown spots on the thorax (*arrow*); **c** three black bands on the tibia (*arrow*); **d** trident-shaped mark on the dorsal part of the abdomen (*arrow*)

## Data Availability

The datasets supporting the conclusions of this article are included in the article. Sequences obtained during the current study are available from the GenBank database (accession numbers MN719908, MN719912, MN719913 and MN719914).
