# Supplementary figures and images for: A first report of Thelazia callipaeda infection in Phortica okadai and wildlife in national nature reserves in China
Source: Parasit Vectors. 2021 Jan 6;14:13. doi: 10.1186/s13071-020-04509-0 (PMC7789172; doi:10.1186/s13071-020-04509-0)

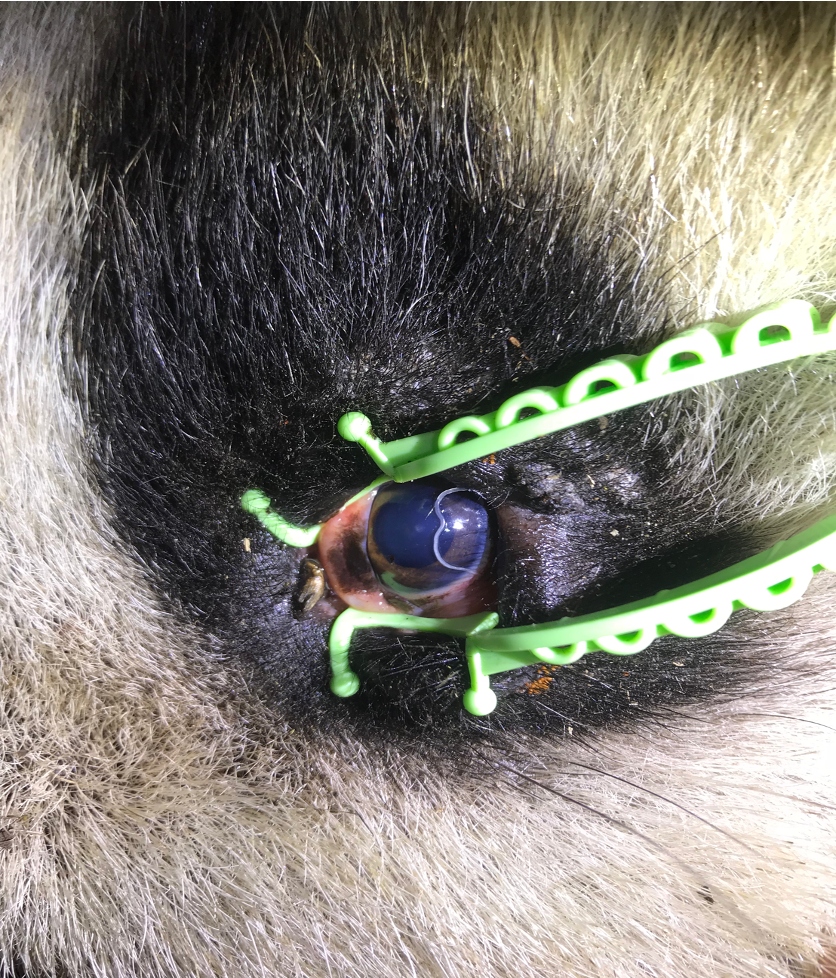


Figure S1


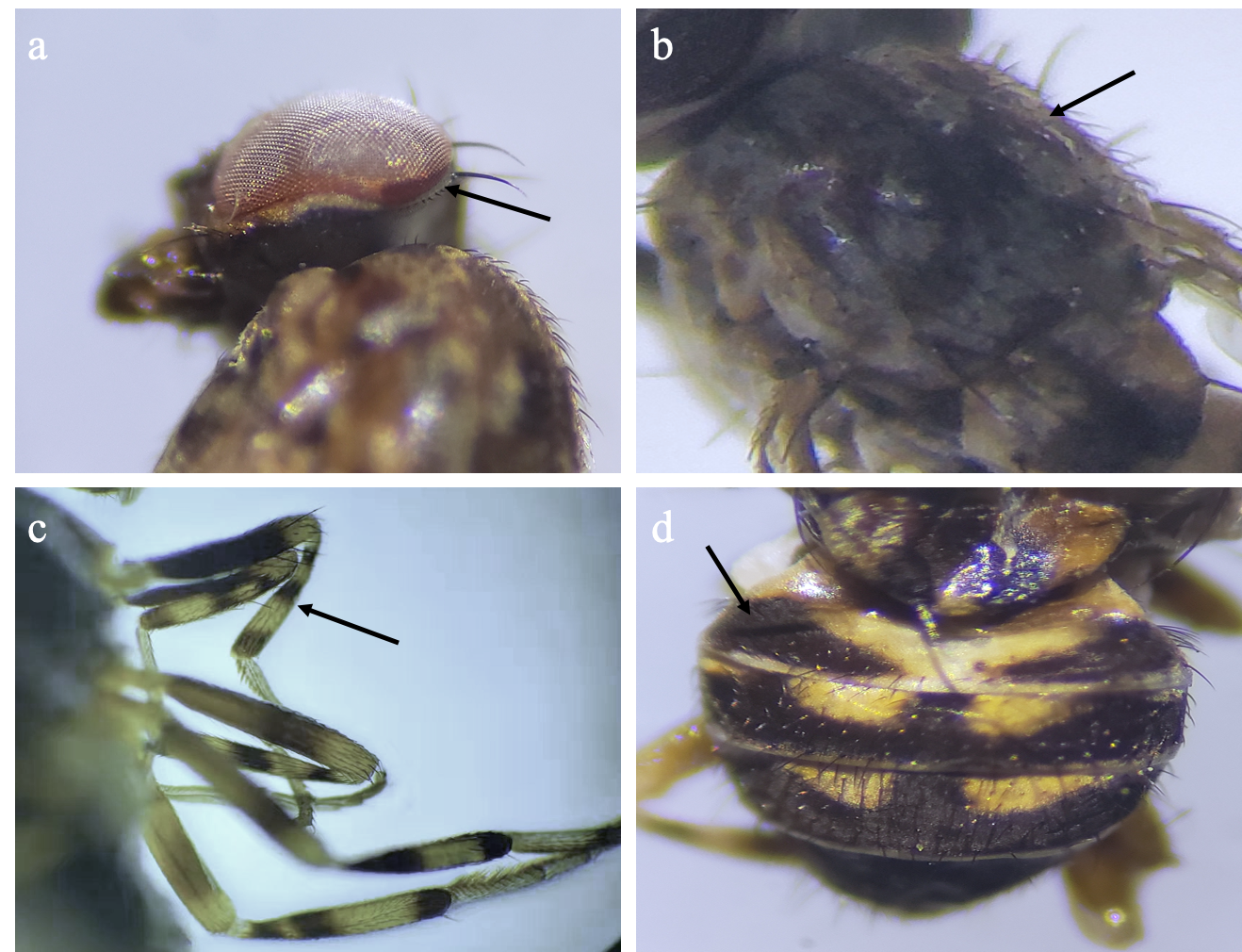


Figure S2

Supplement: Supplementary file 1 — Additional file 1: Figure S1.Thelazia callipaeda in the eye of a giant panda. Figure S2. Light micrographs of Phortica okadai. a White band around the compound eye (arrow); b multiple dark brown spots on the thorax (arrow); c three black bands on the tibia (arrow); d trident-shaped mark on the dorsal part of the abdomen (arrow) [file 13071_2020_4509_MOESM1_ESM.docx]
